# Supplementary material for: MICOM: Metagenome-Scale Modeling To Infer Metabolic Interactions in the Gut Microbiota
Source: mSystems. 2020 Jan 21;5(1):e00606-19. doi: 10.1128/mSystems.00606-19 (PMC6977071; doi:10.1128/mSystems.00606-19)
Supplement: TEXT S1 [file mSystems.00606-19-s0001.pdf]

Supplemental Text S1

December 3, 2019

1 Effect of regularization on growth rates

We consider a community model with growth rates $\mathbf{g} = \{g_1, \dots, g_n\}, g_i \geq 0$ and relative abundances $\mathbf{a} = \{a_1, \dots, a_n\}$. The maximal community growth rate is given by $\mu_c^* = \max \mathbf{a} \cdot \mathbf{g}$ again and we will apply a tradeoff parameter $\alpha \in (0, 1]$.

During regularization we will minimize a regularization function $r(\mathbf{g}) \in \mathbb{R}^+$ with the previously described tradeoff condition:

$$\mu_c = \mathbf{a}^T \mathbf{g} \geq \alpha \mu_c^*$$

If the regularization function is strictly monotonic in $\mathbf{g}$ this inequality will be an equality. In general, finding an analytical solution for the full set of equality and inequality constraints is difficult and requires the evaluation of complementary slackness of the inequalities. This usually prohibits a closed form solution and requires numerical optimization. However, as an approximation, one can find an optimal solution that only considers the optimality constraint and the regularization term and which describes the best case solution when all other constraints can be met.

Thus, it describes the pressure on growth rates due to the regularization and not due to other feasibility constraints.

This can be achieved with a Lagrange multiplier which induces the following minimization problem:

$$\min_{\mathbf{g},\lambda} L(\mathbf{g}, \lambda) = r(\mathbf{g}) - \lambda \left(\mathbf{a}^T \mathbf{g} - \alpha \mu_c^* \right)$$

In general we will only consider the non-trivial case where there exists at least one i such that $g_i > 0$.

1.1 Linear regularization

If r is linear it can be expressed as $r(\mathbf{g}) = \boldsymbol{\beta}^T \mathbf{g} + c$. This induces the following Lagrangian optimization problem:

$$\min_{\mathbf{g},\lambda} L(\mathbf{g}, \lambda) = \boldsymbol{\beta}^T \mathbf{g} + c - \lambda \left(\mathbf{a}^T \mathbf{g} - \alpha \mu_c^* \right)$$

with the following conditions for the existence of a minimum:

$$\forall i : \frac{\partial L}{\partial g_i} = \beta_i - a_i\lambda = 0$$
$$\mathbf{a}^T\mathbf{g} - \alpha\mu_c^* = 0$$

Thus we have to fulfill:

$$\forall i : \lambda = \frac{\beta_i}{a_i}$$
$$\mathbf{a}^T\mathbf{g} = \alpha\mu_c^*$$

As we can see the first equation is independent of g_i and only imposes conditions on the ratio between coefficients and abundance whereas the second condition can be fullfilled by many different sets of growth rates. So linear regularization either has no defined minimum or infinitely many minima. In particular it does not guarantee that $a_i > 0 \iff g_i > 0$.

1.2 Quadratic regularization

We will now consider a simple quadratic regularization (L2) with $r(\mathbf{g}) = \mathbf{g}^T\mathbf{g}$. This induces the following Lagrangian optimization problem:

$$\min_{\mathbf{g},\lambda} L(\mathbf{g}, \lambda) = \mathbf{g}^T\mathbf{g} - \lambda\left(\mathbf{a}^T\mathbf{g} - \alpha\mu_c^*\right)$$

with the following conditions for the existence of a minimum:

$$\forall i : \frac{\partial L}{\partial g_i} = 2g_i - a_i\lambda = 0$$
$$\mathbf{a}^T\mathbf{g} - \alpha\mu_c^* = 0$$

Thus we have to fulfill:

$$\forall i : g_i = \frac{\lambda}{2}a_i$$
$$\mathbf{a}^T\mathbf{g} = \alpha\mu_c^*$$

Plugging the first into the second equation gives:

$$\frac{\lambda}{2}\mathbf{a}^T\mathbf{a} = \alpha\mu_c^*$$
$$\rightarrow \lambda = \frac{\alpha\mu_c^*}{\mathbf{a}^T\mathbf{a}}$$

Pluggin this into the previous equation for g_i now yields:

$$\forall i : g_i = \frac{\alpha\mu_c^*}{\mathbf{a}^T\mathbf{a}}a_i$$

Thus, we have a well defined minimum, and since $\frac{\alpha\mu_c^*}{\mathbf{a}^T\mathbf{a}}$ is a constant, growth rates depend linearly on the abundances and it follows that $a_i > 0 \iff g_i > 0$.

1.3 Conclusions

Thus, in the unconstrained case L2 regularization will yield growth rates that are perfectly correlated with abundances. However, based on additional feasibility constraints, especially resource limitation those optimal growth rates may not be reached which can result in different optima.
