# Supplementary figures and images for: MICOM: Metagenome-Scale Modeling To Infer Metabolic Interactions in the Gut Microbiota
Source: mSystems. 2020 Jan 21;5(1):e00606-19. doi: 10.1128/mSystems.00606-19 (PMC6977071; doi:10.1128/mSystems.00606-19)

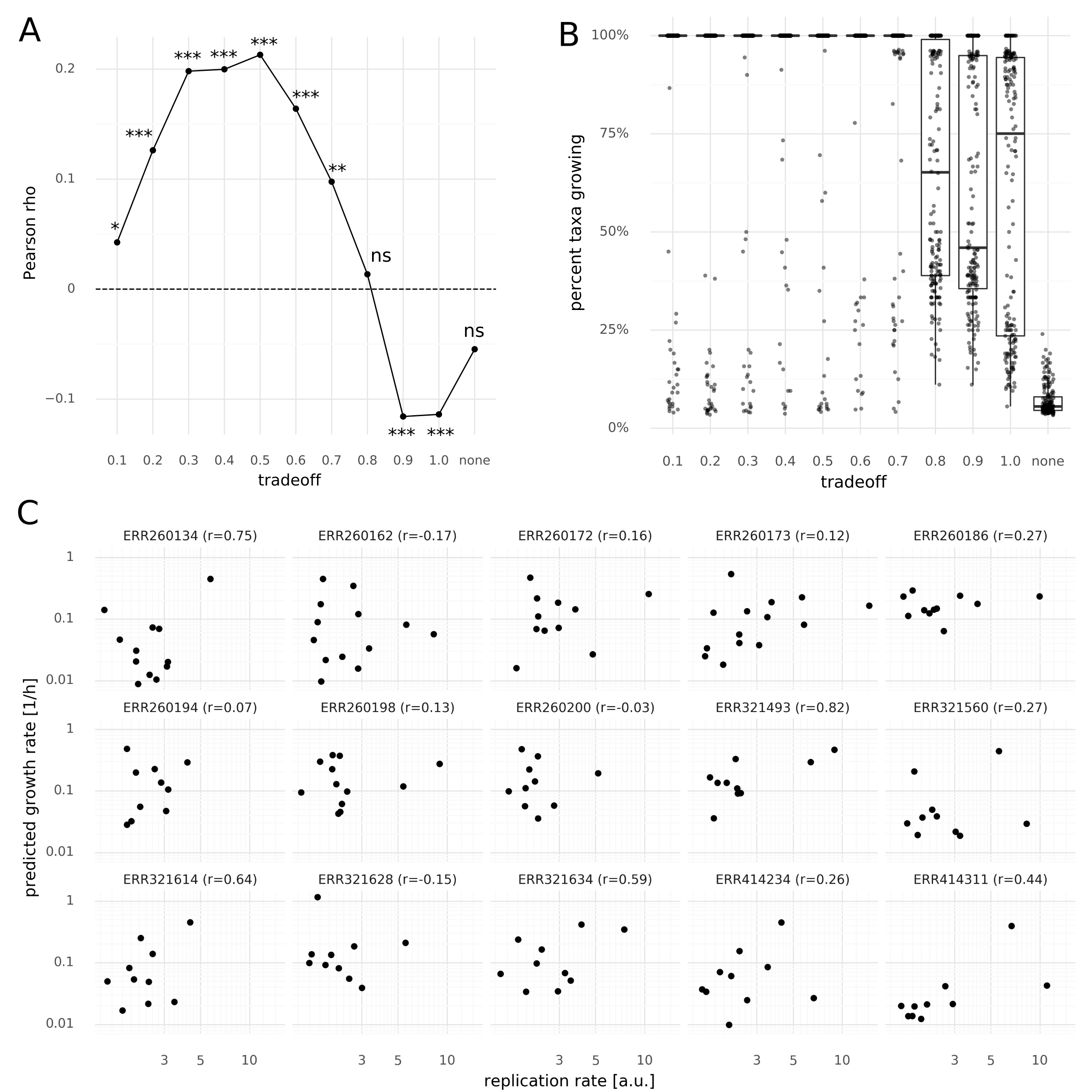

Supplement: FIG S1 [file mSystems.00606-19-sf001.tif]

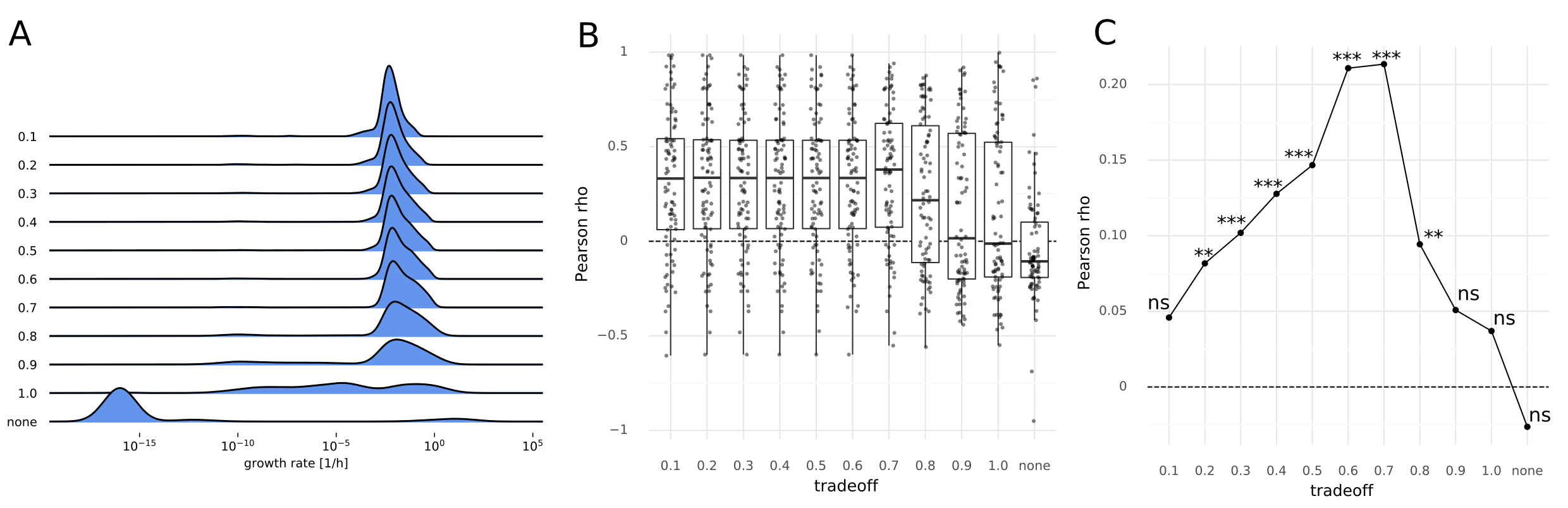

Supplement: FIG S2 [file mSystems.00606-19-sf002.tif]

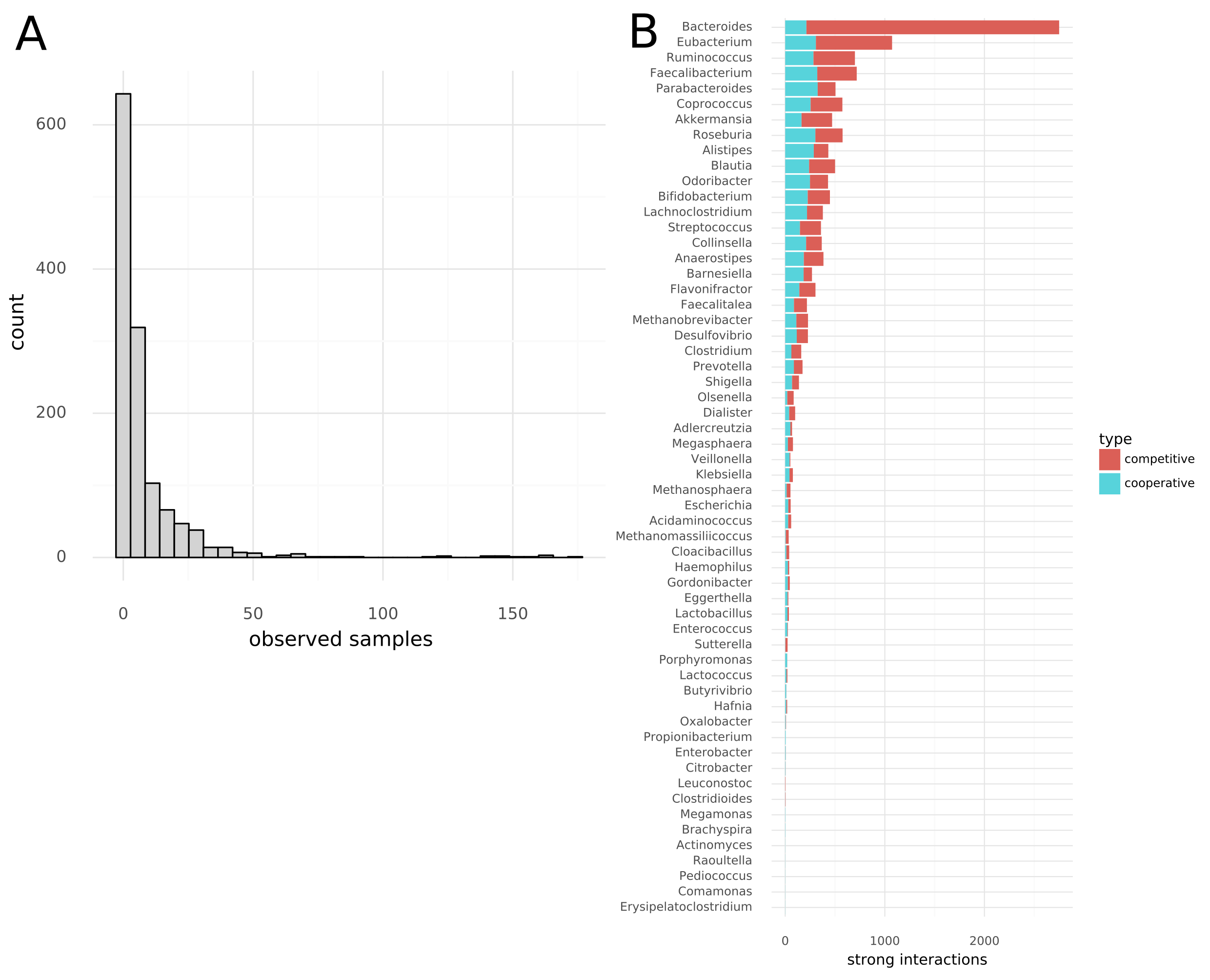

Supplement: FIG S3 [file mSystems.00606-19-sf003.tif]

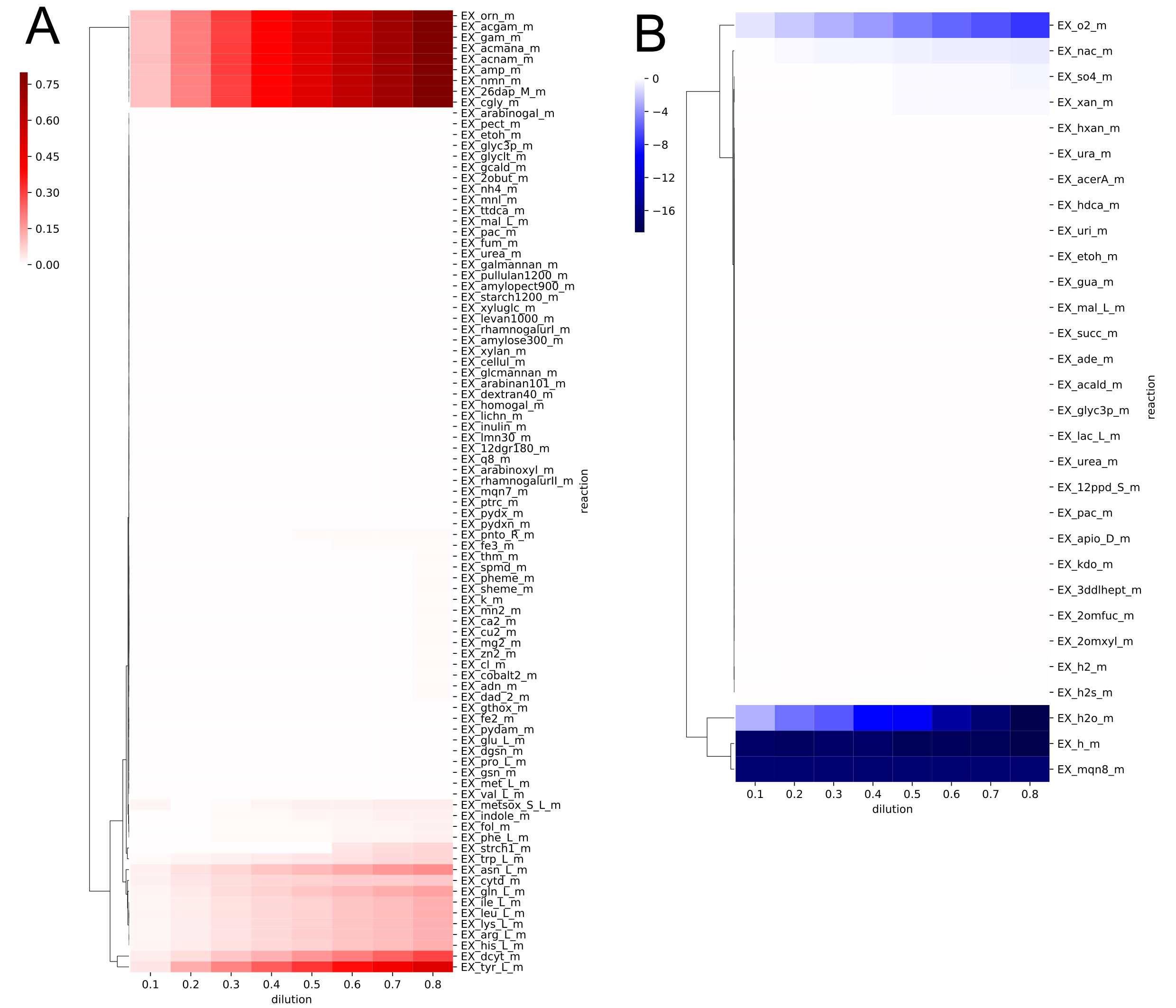

Supplement: FIG S4 [file mSystems.00606-19-sf004.tif]

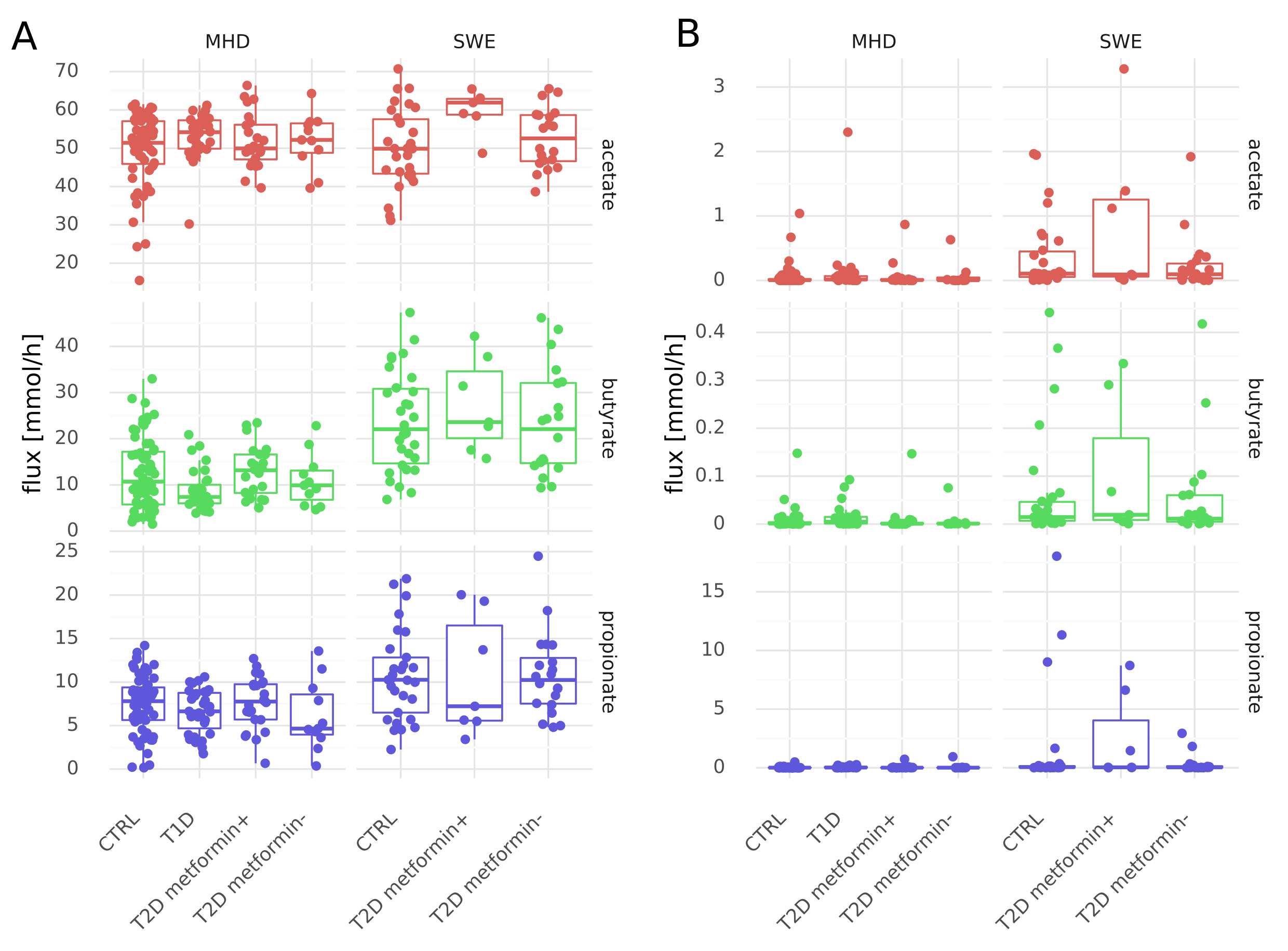

Supplement: FIG S5 [file mSystems.00606-19-sf005.tif]
